# Variations in cyclin D1 levels through the cell cycle determine the proliferative fate of a cell

**DOI:** 10.1186/1747-1028-1-32

**Published:** 2006-12-18

**Authors:** Ke Yang, Masahiro Hitomi, Dennis W Stacey

**Affiliations:** 1 The Department of Molecular Genetics, The Lerner Research Institute, The Cleveland Clinic Foundation, 9500 Euclid Ave. Cleveland OH, 44072, USA

## Abstract

We present evidence that variations in cyclin D1 levels through the cell cycle are essential for continuing proliferation. Cyclin D1 levels must be high during G1 phase for a cell to initiate DNA synthesis, but then must be suppressed to low levels during S phase to allow for efficient DNA synthesis. This suppression during S phase is apparently regulated by cell cycle position alone and occurs automatically during each cell cycle. If the cell is to continue proliferating, cyclin D1 levels must be induced once again during G2 phase. This induction depends upon the activity of proliferative signaling molecules, and ensures that the extracellular environment continues to be conducive for growth. We propose that the suppression of cyclin D1 levels during each S phase ensures that the subsequent induction during G2 phase, and the resulting commitment to continuing proliferation, is closely linked to the cellular growth environment.

## Background

Cyclin D1 plays a central role in the regulation of proliferation, linking the extracellular signaling environment to cell cycle progression [1]. The expression level of cyclin D1 is highly responsive to the action of proliferative signals including growth factor receptors, Ras, and their downstream effectors. Regulation in expression level involves a variety of mechanisms including production, stability and utilization of cyclin D1 mRNA; as well as protein stability, localization, and association. Its expression increases upon stimulation of quiescent cells to enter the cell cycle, while it has been proposed to shuttle in and out of the nucleus through the cell cycle of actively cycling cells [2]. Once the expression level of cyclin D1 is determined as the sum result of the cellular signaling environment, it binds cyclin dependent kinase 4 or 6 (CDK4/6) to form an active kinase for the retinoblastoma protein (Rb). The growth inhibitory action of Rb is neutralized following phosphorylation by cyclin D1/CDK4, to allow E2F transcription factors to promote the transcription of genes required for cell cycle progression [3]. Cell cycle progression ceases following neutralization of cyclin D1 by microinjected antibodies in many cultured cells [4,5], even though knockout studies indicate that many of its functions can be performed by other cyclins during development [6-8].

## Cyclin D1 levels vary through the cell cycle

**Figure 1:**
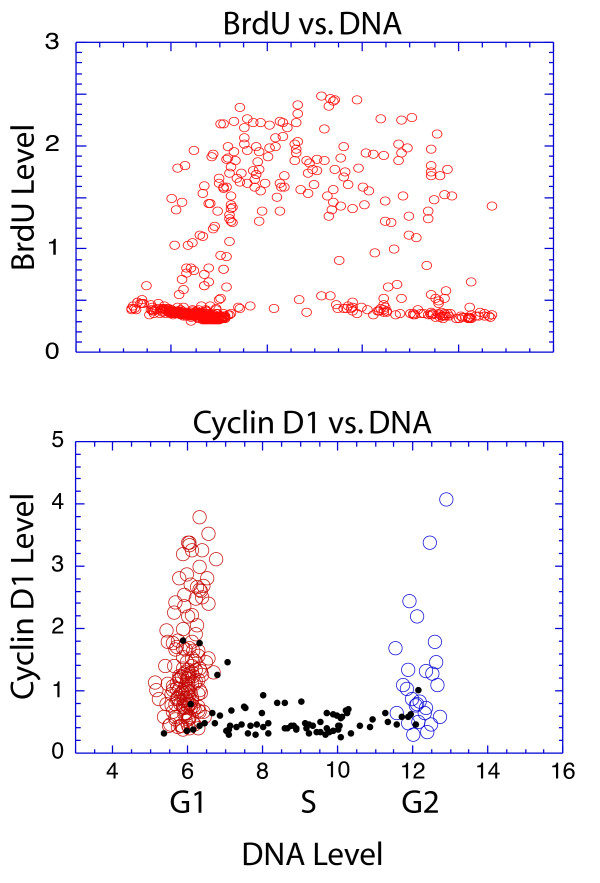
Profile of BrdU and cyclin D1 expression through the cell cycle. MRC5, human diploid fibroblasts, were pulsed with BrdU for 20 min, fixed and stained for cyclin D1 and BrdU with fluorescent antibodies, while DNA was stained with DAPI. Images of each fluorochrome were subjected to image analysis to quantitate the level of each fluorochrome in each cell. (Top) BrdU levels are plotted vs. DNA content for each cell; and (Bottom) cyclin D1 levels are plotted vs. DNA content. BrdU negative cells in G1 (red), or G2 (blue) phases are indicated by open circles; while S phase cells (black) are represented by smaller closed circles.

**Figure 2:**
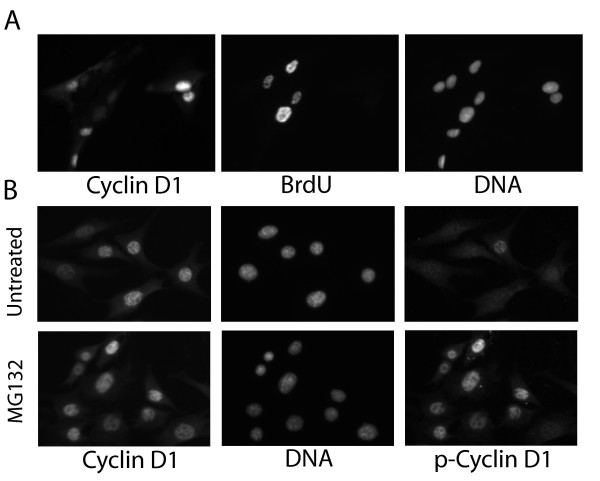
Cyclin D1 and phospho-cyclin D1 staining patterns. (A) MRC5 cells were pulsed with BrdU and stained as described in Figure 1. Fluorescence photographs of BrdU, cyclin D1 and DNA stains for a single group of cells are presented. Separate cells stain for cyclin D1 and BrdU. (B) NIH3T3 cells were fixed and stained with antibodies against total cyclin D1, or cyclin D1 phosphorylated on Thr-286. This procedure was performed on untreated cells, or following a three hr treatment with MG132 to block proteasomal degradation. Fluorescence images of the same area of cells are presented, along with DAPI stained DNA. No accumulation of cytoplasmic cyclin D1 or phospho-cyclin D1 is apparent in any of these cells.

**Figure 3:**
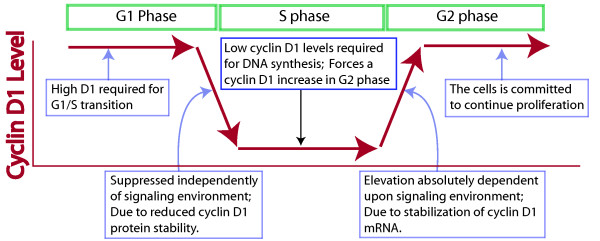
Diagram of cyclin D1 expression through the cell cycle. The bold red line indicates the expression profile of cyclin D1 through the cell cycle of a normal cell. The importance of this expression pattern, along with an explanation of how the changes are regulated is included.

The central role of cyclin D1 in promoting entry into the cell cycle suggests it should also be important in regulating cell cycle progression once it has initiated, although this aspect of its action has been poorly understood due to the difficulty of studying cells that are actively cycling. In order to study the function of cyclin D1 in cells actively progressing through the cell cycle, we developed a quantitative image analysis technique for protein quantitation in individual cells. Rather than attempt to force all cells into a single cell cycle position, this approach allowed the identification of the cell cycle position of individual cells within an asynchronous culture. An asynchronous culture was pulsed with BrdU and fixed, then the BrdU, DNA, and cyclin D1 were stained with fluorescent labels. Accurate quantitation allowed the determination of the level of each of these markers within each individual cell. Cell cycle position was determined based upon DNA content and BrdU staining. Cyclin D1 expression could then be quantitated for cells in all cell cycle periods simultaneously (Fig. 1). Importantly, cyclin D1-associated fluorescence in stained cells was shown to be directly proportional to total cyclin D1 protein as judged by western analysis in synchronized cultures [9]. Because the technique relied upon microscopic examination of monolayer cells, it was possible to quantitate the levels of nuclear antigens with a high degree of accuracy as indicated by the detailed profile of the BrdU stain vs. DNA content (Fig. 1, top). It is also possible to manipulate individual cells with microinjection techniques prior to analysis. With this approach we observed high levels of cyclin D1 in G1 and G2 phase cells. Cells in S phase, on the other hand, uniformly contained low levels of cyclin D1 (Fig. 1 bottom) [9]. This profile was observed in all normal cells in monolayer culture studied to date, including fibroblasts and epithelial cells. The results were observed with a variety of different antibodies (Fig. 2). Moreover, the reduction in total cyclin D1 protein content during S phase has been observed by western analyses of synchronized cultures, and in stained sections of normal tissues. Based upon the accumulated evidence there is little reason to doubt that cyclin D1 levels in actively cycling cells increase during G2 phase, are maintained through mitosis and G1 phase, and decline when DNA synthesis begins (Fig. 3).

Our first goal was to determine how the increase in cyclin D1 during G2 phase is regulated, and what biological role it plays. This increase in cyclin D1 level was shown to be absolutely dependent upon proliferative signaling and cellular Ras activity, since it was blocked in cells deprived of growth factors, or microinjected with neutralizing anti-Ras antibody. Interestingly, these treatments suppressed cyclin D1 levels in G2 phase cells several hours prior to any observable effect upon cyclin D1 levels during G1 phase [9]. Cyclin D1 levels were apparently determined by extracellular signaling during G2 phase, and then maintained at a relatively uniform level until DNA synthesis began. In direct support of this notion, when oncogenic Ras protein was microinjected into actively cycling cells it had effects upon cellular migration and gene activation throughout the cell cycle, even though it was able to promote cyclin D1 accumulation only during G2 phase [10]. We conclude that proliferative signaling controls the elevation of cyclin D1 levels during G2 phase (Fig. 3).

## Elevation of cyclin D1 during the G2 phase promotes continuing proliferation

In order to understand why the elevation of cyclin D1 during G2 phase is so important, it is necessary to consider the results of an unrelated series of experiments. We found that when anti-Ras antibody was microinjected into actively cycling cells, it efficiently blocked cell cycle progression, but only if present during G2 phase [5]. Obviously, a target of Ras activity that is specifically active during G2 phase is required to allow the cell to continue active proliferation. As direct evidence that this Ras target is cyclin D1, we found that when Ras activity was neutralized by microinjected anti-Ras antibody, the cell could continue proliferating into the next cell cycle so long as cyclin D1 levels were artificially elevated [11,9]. We conclude that the ability of proliferative signaling to induce cyclin D1 levels specifically during G2 serves to commit the cell to continue active cell cycle progression. Importantly, this critical decision to continue active proliferation takes place just prior to entry into mitosis. In this way, at the completion of mitosis the cell can immediately be directed either to continued proliferation or entry into quiescence, depending upon its extracellular growth environment during the preceding G2 phase (Fig. 3). The stimulation of cyclin D1 levels during G2 phase relies on the ability of mitogen to stabilize its message, although cell cycle-dependent changes in protein stability also play a role in this G2 phase increase in cyclin D1 levels [12,13]. In support of this conclusion, cyclin D1 mRNA was shown to be continuously synthesized and relatively stable until serum was removed from the culture, at which time its stability was dramatically and immediately reduced [12].

## Cyclin D1 suppression during S phase ensures constant proliferative signaling

We postulated that cyclin D1 levels are suppressed during S phase to ensure that proliferation continues only in cells with a positive growth environment during the succeeding G2 phase. In other words, the suppression of cyclin D1 levels during S phase effectively erases the effects of any signaling events from previous cell cycle periods, and ensures that for continued proliferation the cellular environment must be reassessed prior to the initiation of every new cell cycle. To gain further evidence for this idea we undertook an analysis of the mechanism by which cyclin D1 levels are suppressed during S phase. Metabolic labeling studies and quantitative image analysis demonstrated that the decline of cyclin D1 during S phase was due to a decrease in protein stability specifically during S phase in actively cycling cells [13]. Further studies demonstrated that cyclin D1 declined as the result of proteasomal degradation following phosphorylation of Thr-286 [14]; since mutants of this site maintained high cyclin D1 levels through S phase [13,15], and accumulation of phosphorylated cyclin D1 increased most rapidly in S phase cells when proteasomal degradation was inhibited [13].

Efforts were next made to identify the kinase involved in phosphorylation of Thr-286 as a means of studying the molecular mechanism of cyclin D1 suppression, and thereby its biological regulation. The critical regulatory kinase, glycogen synthase kinase 3 (GSK3), has been shown to phosphorylate cyclin D1 on Thr-286 in vitro, and is postulated to regulate cyclin D1 levels and intracellular distribution [16]. GSK3 is inhibited following phosphorylation by AKT, which is in turn activated by phosphatidylinositol-3 kinase (PI3 kinase); a prime mediator of proliferative signaling. Thus, proliferative signaling would stabilize cyclin D1 by stimulating the PI3 kinase/AKT pathway to phosphorylate and inactivate GSK3. The inactive GSK3 would then be unable to phosphorylate and promote the degradation of cyclin D1. If this were to account for the suppression of cyclin D1 during S phase, it would be necessary that proliferative signaling, or its connection to GSK3, cease only during S phase.

## GSK3 does not regulate cyclin D1 levels

To test this model our goal was to devise means to identify an alteration in proliferative signaling specifically during S phase. The possibility that such an alteration might be observed was supported by the fact, as mentioned above, that oncogenic Ras, while active throughout the cell cycle, is able to promote cyclin D1 elevation only during G2 phase [10]. Moreover, we have shown that proliferative signaling is able to suppress p27 levels throughout the cell cycle, but that different signaling pathways lead to this suppression during each cell cycle period [17]. Thus, it appeared reasonable to assume that an alteration in signaling activity or its targets might effect GSK3 differentially through the cell cycle. This, however, was not observed. The cell cycle dependent change in PI3 kinase activity was assessed by expression of a green fluorescent protein linked to a protein (the PH domain) with the ability to move to the membrane and bind the lipid product of PI3 kinase. PI3 kinase activity, as identified by movement of this marker protein to the plasma membrane, was observed only following removal and re-addition of serum growth factors to either a quiescent or an actively cycling culture. There was no evidence of cell cycle related changes in PI3 kinase activity. As another mechanism to measure activity of the PI3 kinase pathway, we studied synchronized cultures and found no evidence for alterations in AKT or GSK3 activity through the cell cycle, even though cyclin D1 levels fell during S phase and increased during G2 phase in these same cells. Finally, we reasoned that if alterations in cyclin D1 levels through the cell cycle were produced by alterations in the activity or effects of proliferative signaling, then global inhibition of proliferative signaling would tend to eliminate the cell cycle dependent changes in cyclin D1 levels. As noted above, however, the removal of serum, or the inhibition of Ras activity did not eliminate cell cycle related changes in cyclin D1 levels; rather, these treatments specifically reduced cyclin D1 levels during G2 phase without effecting G1 phase levels. Moreover, in cells transformed by oncogenic Ras, where proliferative signaling has been shown to be constant through the cell cycle, the decline in cyclin D1 levels during S phase is still observed. We concluded that cell cycle related alterations in proliferative signaling are not likely to be responsible for the suppression of cyclin D1 during S phase (Fig. 3) [18].

The above results not only raise questions regarding the role of GSK3 in the suppression of cyclin D1 during S phase, they raise questions regarding the potential role of GSK3 in the regulation of cyclin D1 levels in general. To address this concern GSK3α and GSK3β activity were both inhibited by a variety of chemical inhibitors, and protein levels of both proteins were suppressed with siRNA, without any observable alteration in cyclin D1 expression characteristics. Moreover, a mutation in the site normally phosphorylated by AKT renders the GSK3β protein constitutively active. A plasmid expressing such an activated mutant was introduced into cultured cells by microinjection. This resulted in a dramatic increase in the overall levels of GSK3 activity, but once again without any alteration in the expression level of cyclin D1 or its level of phosphorylation on Thr-286 in any cell cycle phase. These observations suggested that the action of GSK3 within the fibroblast cells we were studying did not directly influence cyclin D1 levels. To directly confirm this fact an experiment was performed to demonstrate the differential effects of GSK3 inhibition upon cyclin D1 and β-catenin phosphorylation and degradation. β-catenin is phosphorylated by GSK3 bound in a multi-protein complex. The ability of GSK3 to phosphorylate β-catenin is inhibited by signaling through the Wnt pathway, resulting in a decreased rate of β-catenin phosphorylation. Thus, the inhibition of GSK3 activity can be assessed by a decrease in β-catenin phosphorylation. NIH3T3 cells were treated with varying levels of LiCl, a potent and relatively specific inhibitor of GSK3. The levels of phosphorylated β-catenin and of phosphorylated cyclin D1 were simultaneously analyzed in the treated cells. As expected, the levels of phosphorylated β-catenin were reduced by the LiCl treatment. Of critical importance however, the levels of phosphorylated cyclin D1 were not altered in these cells, even though it was clear that GSK3 had been inhibited [18]. It is also critical to note that there was no apparent redistribution of cyclin D1 between nucleus and cytoplasm in these treated cells (Fig. 2) [19].

We conclude that in these actively cycling cells the expression level of cyclin D1 is not regulated by, and not a physiological substrate of GSK3. We initiated these studies to understand the biological mechanism by which cyclin D1 is suppressed during S phase, as a means of understanding its physiological significance. Our results not only eliminate the involvement of GSK3, these results suggest that proliferative signaling in general is not involved in suppression of cyclin D1 during S phase. This suppression apparently takes place automatically upon the initiation of DNA synthesis, regardless of the signaling environment of the cell (Fig. 3).

## Biological importance of cyclin D1 expression through the cell cycle

The fact that proliferative signaling is not apparently involved in the suppression of cyclin D1 during S phase has important implications. While the critical kinase involved is not known, it appears likely that it will be regulated not so much by the signaling environment of the cell as by the fact that the cell has entered S phase. Thus, the suppression of cyclin D1 during S phase appears to be governed by cell cycle position, resulting in the suppression of cyclin D1 during each S phase. An understanding of why cyclin D1 must be suppressed during S phase comes from previously reported studies that indicate that cyclin D1 has the ability to inhibit DNA synthesis by virtue of its ability to bind the critical regulator of DNA synthesis, PCNA [20-22]. In support of this conclusion, the level of exogenous cyclin D1 expression was directly linked to an increase in the length of S phase [23,18], and of the entire cell cycle [18]. Thus, not only does the cell automatically suppress cyclin D1 during S phase; this suppression appears essential to efficient cell cycle progression. These two facts clearly support our initial proposal that the reduction of cyclin D1 during S phase enforces the requirement for continuing positive growth conditions. In other words, the suppression of cyclin D1 during S phase serves as an automatic reset to erase the effects of proliferative signaling from any previous cell cycle period, and ensure that the cell reassess its proliferative environment prior to committing to continued active proliferation during G2 phase (Fig. 3).

The fact that cyclin D1 levels must be suppressed during S phase might also limit tumor formation directly. Any mutation or alteration resulting in high cyclin D1 levels might promote uncontrolled passage through G1 phase and into DNA synthesis regardless of growth conditions. The resulting cyclin D1 levels, however, might be so high as to block progression through S phase. Thus, for active proliferation, it is likely that the expression of this critical regulator of growth be within a limited expression range [24]. The ability of alterations in cyclin D1 expression to play a role in tumor formation, therefore, might require alterations in the processes described above that normally limit its permissible expression range. In support of this contention, we have observed altered cell cycle expression profiles in many of the tumors and tumor cell lines we have analyzed (manuscript in preparation).

## Other considerations

Because cyclin D1 suppression during S phase is apparently so critical in its overall ability to regulate cell cycle progression, it is logical to question why this suppression is not more universally recognized. A model suggesting the decline of cyclin D1 during S phase as the result of displacement by p16Ink4 was proposed a number of years ago [1]. In addition, cyclin D1 suppression during S phase is supported by FACS data [25,26], along with biochemical analyses of cells separated into cell cycle positions by elutriation [27], or synchronized in mitosis [4], or by serum addition [28-30]. Unfortunately, cell cycle synchrony is often lost following mitogen stimulation of quiescent cells prior to exit from S phase, so that it is difficult to observe a biochemical fall in cyclin D1 levels in quiescent cells stimulated with serum. FACS analysis is complicated by the fact that cyclin D1 is a rather weak, nuclear antigen. Finally, as noted above, cyclin D1 levels do not decline during S phase in many tumor cells, so that only studies of normal cell types can with certainty be used to analyze cyclin D1 expression through the cell cycle.

There is no question from the literature that cyclin D1 is reduced in the nucleus of many cell types during S phase [28,4], based upon fluorescence staining (Fig. 2A). This has been explained in the past, however, as the result of intracellular redistribution following phosphorylation by GSK3 [31-33]. Exit of cyclin D1 from the nucleus into the cytoplasm would potentially mask its activity by separating it from its critical substrates, thus eliminating its ability to inhibit DNA synthesis. On the other hand, if the elimination of cyclin D1 from the cell serves as the means to ensure the constant presence of conducive growth conditions as proposed above, the simple separation from substrates might not have the profound regulatory consequences that total elimination from the cell would have. For this reason, it is of critical importance to clearly distinguish between redistribution and degradation. If phosphorylation of cyclin D1 on Thr-286 is responsible for nuclear export and subsequent degradation, we reasoned that the low levels of phosphorylated cyclin D1 in normal cells would be cytoplasmic. Moreover, if proteasomal degradation is blocked and the levels of total and phosphorylated cyclin D1 increase, we reasoned that a significant proportion of the excess total cyclin D1, and the majority of the phosphorylated cyclin D1 awaiting proteasomal degradation would localize to the cytoplasm. To test these possibilities, NIH3T3 cells were fixed and stained for total cyclin D1, cyclin D1 phosphorylated on Thr-286, and for DNA. Cells were analyzed with or without a three hr MG132 treatment to block proteasomal activity. In all cells tested, total cyclin D1 and that phosphorylated on Thr-286 was localized to the nucleus (Fig. 2B). This conclusion was clearly apparent following proteasomal inhibition, although only a small number of untreated cells express identifiable phospho-cyclin D1. Even in these few unreated cells, however, the increase in phospho-cyclin D1 staining was apparently always localized to the nucleus (although the background cytoplasmic staining makes this conclusion uncertain, Fig. 2B). These results are not consistent with a model in which cyclin D1 moves to the cytoplasm to await degradation, particularly if that degradation requires phosphorylation on Thr-286.

It is important to emphasize that the conclusion that cyclin D1 or any nuclear protein has exited from the nucleus into the cytoplasm is extremely difficult to firmly establish. If this conclusion is made on the basis of subcellular localization following cellular disruption, the determination becomes more a matter of nuclear binding than localization [26], since a protein as small as cyclin D1 might be released from the nucleus during its purification. If the determination of cellular localization relies simply upon analysis of stained images as described above, the challenge becomes even greater. The cross section of the nucleus in monolayer cells is often a small fraction of the entire cell; and this fraction varies dramatically from one cell to another. Moreover, the nonspecific staining of most antibodies, including many anti-cyclin D1 antibodies, is greater in the cytoplasm than the nucleus. Therefore, it is difficult to prove that an antigen has left a small nucleus and redistributed into a larger cytoplasm, where variations in cell thickness and size complicate accurate determination of background staining. Such a conclusion would require accurate determination of background staining for the given cell, the careful quantitation of increases of fluorescence above that background, and a determination of whether or not this increase could be due to the decrease in staining in the nucleus. In our careful, quantitative analyses of this type we have found no evidence of export form the nucleus to the cytoplasm [9]. Moreover, following introduction of green fluorescent protein tagged cyclin D1, we have found that cytoplasmic fluorescence is always indicative of a dramatic over expression of exogenous cyclin D1. Similar conclusions result from staining of cells following elevated expression of exogenous, unmodified cyclin D1. We find no evidence for exit of cyclin D1 from the nucleus into the cytoplasm as a cell cycle regulated event. Our studies do not in every detail duplicate the growth conditions and cell types others have used, but we are confident that in our cells under the conditions described above, cyclin D1 is degraded during S phase. This fact is supported by the biochemical studies we and others have performed as indicated above [13,4,27-30].

A final consideration regards the potential role of GSK3 in the control of cell growth in general. In *Drosophila *PI3 kinase signaling inactivates the GSK3 analogue as in mammalian cells. In flies engineered to have a GSK3 analogue which cannot be inhibited by PI3 kinase signaling, however, the animals grow normally; indicating that targets in addition GSK3 must be involved in the ability of PI3 kinase to regulate cell growth [34]. Similar conclusions have been reached in genetic studies of mice where neither development, nor cell growth and proliferation were effected by blocking the activation of either GSK3α or GSK3β [35]. In bone growth and development, where GSK3 activity does appear to play a role, it does so by regulating the production of a growth factor rather than by directly regulating proliferation [36]. These data support our observation that GSK3 does not play a major role in cell cycle regulation in actively proliferating cells. None of these results, however, exclude the possibility that GSK3 might play an important role in the proliferation of other types of cells, or cells in the absence of proliferative signaling.

*In conclusion*, the evidence indicates that cyclin D1 levels not only regulate the initiation of cell cycle progression in quiescent cells, but that they play a critical role in the decision of a cell to continue proliferating. This decision is made during G2 phase when proliferative signaling induces an increase in cyclin D1 levels. The apparently automatic decline in cyclin D1 levels during the preceding S phase allows efficient DNA synthesis, and ensures that proliferative conditions are conducive for continued growth at the time of commitment for continuing proliferation during G2 phase (Fig. 3). We find no evidence that GSK3 is involved in regulating cyclin D1 expression or intracellular localization in actively proliferating cells.

## Competing interests

The author(s) declare that they have no competing interests.

## Authors' contributions

Each of the author contributed to the preparation of this review, and were involved in the experiments reported previously upon which it is based.
